# Identification of novel small chemical compounds that inhibit Ebola virus VP40-mediated virus-like particle production

**DOI:** 10.1016/j.virusres.2026.199684

**Published:** 2026-01-03

**Authors:** Tomoko Tsuruta, Satoshi Mizuta, Ayako Izumisawa, Jiro Yasuda, Shuzo Urata

**Affiliations:** a Department of Medical and Applied Virology, National Research Center for the Control and Prevention of Infectious Diseases (CCPID), Nagasaki University, 1-12-4 Sakamoto, Nagasaki, 852-8523, Japan; b Center for Bioinformatics and Molecular Medicine, Graduate School of Biomedical Sciences, Nagasaki University, Nagasaki, Japan; c Department of Emerging Infectious Diseases, National Research Center for the Control and Prevention of Infectious Diseases (CCPID), Nagasaki University, 1-12-4 Sakamoto, Nagasaki, 852-8523, Japan; d Department of Emerging Infectious Diseases, Institute of Tropical Medicine (NEKKEN), Nagasaki University, 1-12-4 Sakamoto, Nagasaki, 852-8523, Japan

**Keywords:** Ebola virus, VP40, Chemical compound, Particle production

## Abstract

The central role of Ebola virus (EBOV) VP40 in nascent virion assembly and budding from infected host cells makes it an important therapeutic target. Previously, we reported two novel chemical compounds (NUSU#1 and NUSU#2) that inhibit EBOV VP40-mediated virus-like particle (VLP) production. In this study, we aimed to develop and identify novel synthetic compounds derived from NUSU#1. Forty-five compounds were synthesized and examined for their effects *in vitro*. Through cell viability and screening assays that we previously developed and reported, five novel synthesized compounds were selected for their inhibitory effects on EBOV VP40-mediated VLP production. Although none of them showed a stronger inhibitory effect on VLP production than NUSU#1, four of the five selected compounds exhibited a statistically significant reduction in EBOV VP40-mediated VLP production compared with the control treatment using a VLP assay.

## Research Highlight

• Forty-five chemical compounds were synthesized that were derived from the NUSU#1.
• Four compounds were identified that inhibited Ebola VLP production.
• Identified four compounds exhibited less cell toxicity than NUSU#1.

## 1 Introduction

Ebola virus disease (EVD) and Marburg virus disease (MVD) are caused by the infection of Ebola virus (EBOV) and Marburg virus (MARV), respectively. Both are transmitted through direct contact with infected people or materials, and has a high fatality rate. Genus *Orthoebolavirus* contains 6 different species, such as *Orthoebolavirus zairense* (EBOV), *Orthoebolavirus sudanense* (Sudan virus, SUDV), *Orthoebolavirus taiense* (Tai Forest virus, TAFV), *Orthoebolavirus bundibugyoense* (Bundibugyo virus, BDBV), *Orthoebolavirus bombaliense* (Bombali virus, BOMV), and *Orthoebolavirus restonense* (RESTV) (Biedenkopf et al., 2023). The genus *Orthomarburgvirus* includes the Marburg and Ravn viruses, which are now found in one species (*Orthomarburgvirus marburgense,* MARV) (Biedenkopf et al., 2023). Both EVD and MVD outbreaks have occurred several times.

Since the first discovery of EBOV in 1976, there have been several outbreaks in Africa, and the outbreak that occurred between 2013 and 2016 resulted in more than 28,000 cases and more than 10,000 deaths (Lo et al., 2017) . To date, two antibody based therapeutics (Ebanga and Inmazeb (El Ayoubi et al., 2024)) and one vaccine (Ervebo (Saphire, 2020) have been approved by the Food and Drug Administration (FDA), but their efficacy is not perfect, and there is concern about the emergence of mutant viruses resistant to these vaccines or therapeutics. In addition to EBOV, BDBV, SUDV, TAFV, and MARV also cause hemorrhagic fever in humans (Biedenkopf et al., 2023; Kuhn Jens H., 2021). Approved vaccine and antibody cocktails are effective only against EBOV and not against other filoviruses. Therefore, developing effective anti-filoviral therapeutics against EBOV and other human pathogenic filoviruses is urgently needed.

EBOV is an enveloped virus with a negative-sense, single-stranded RNA genome (*Mononegavirales*) encoding seven structural proteins (Kuhn Jens H., 2021). Among them, VP40 is known to play an important role in virus particle formation (Jasenosky et al., 2001; Noda et al., 2002; Timmins et al., 2001). Accordingly, the sole expression of filovirus VP40 can produce non-infectious virus-like particle (VLP) by interacting with a variety of host factors such as Tsg101 and E3 ligases through their late (L)-domains (Irie et al., 2005; Licata et al., 2003; Timmins et al., 2003; Urata et al., 2007; Urata and Yasuda, 2010; Yasuda et al., 2003). Therefore, interference with the VP40 function could disrupt EBOV propagation in cells. Small tags, such as the Flag-tag (8 amino acids (aa)) or HA-tag (9 aa), do not interfere with VP40-mediated particle production when tagged at the VP40 N-terminus (Urata et al., 2022). The C-terminus of VP40 is known to recognize the host membrane, including plasma membrane (PM) (Adu-Gyamfi et al., 2013; Soni et al., 2013; Stahelin, 2014), and the attachment of an additional gene at the C-terminus interferes with the VP40-PM interaction, resulting in defects in VLP production. The large firefly luciferase gene (1650 base pairs (bp), 550 aa)-tagged VP40 did not efficiently produce VLPs, most likely due to interference with host factors (McCarthy et al., 2006). This restriction prevented us from developing an efficient screening method targeting the particle production step. Several studies have reported potential VP40 inhibitors using *in silico* methods (Broni et al., 2023; Joob and Wiwanitkit, 2016; Karthick et al., 2016; Khan et al., 2021) with previously reported structures (Bornholdt et al., 2013; Dessen et al., 2000; Gomis-Ruth et al., 2003; Ruigrok et al., 2000). The first report of tagged VP40 for screening was described using a truncated β-lactamase gene (first 72 bp (24 aa, signal peptide) was removed) that was attached to the N-terminus of VP40 to produce VLPs efficiently; however, this study aimed to identify novel chemical compounds for the virus entry step (Kouznetsova et al., 2014). With the appreciation of the development of the smaller luciferase gene, we identified two novel chemical compounds that inhibited EBOV VP40-mediated VLP production using NanoLuciferase (Nluc, 513 bp, 171 aa)-tagged VP40 construct (Lin et al., 2020; Urata et al., 2022). Especially, NUSU#1 inhibited not only EBOV VP40-mediated VLP production but also BDBV, SUDV, TAFV, MARV, and non-human pathogenic Reston virus (RESTV) VP40-mediated VLP production.

This study aimed to identify novel chemical compounds synthesized from NUSU#1 that showed a reduction of EBOV VP40-mediated VLP production *in vitro*.

## 2 Materials and methods

**Synthesis of novel amides, carbamoyl acetamides, and other derivatives from NUSU#1.** Unless otherwise noted, all the starting materials and reagents were obtained from commercial suppliers and used without further purification. All chemicals were purchased from Merck Millipore (Burlington, MA, USA), Nacalai Tesque (Kyoto, Japan), Tokyo Chemical Industry (Tokyo, Japan), and Fujifilm (Osaka, Japan) and were used as received. All the solvents were purchased from Fujifilm. NUEbo 45 was prepared as previously reported (Komai, 1998). Details of the synthesis procedure for the compounds are described in the Supplemental Information.

**Cells and plasmids.** 293T cells (derived from human embryonic kidney, JCRB2202; RIKEN BioResource Research Center (Kyoto, Japan)) were maintained in Dulbecco’s modified Eagle’s medium (044-29765, FUJIFILM) with 10% fetal bovine serum (FBS), 100 IU/mL penicillin, and 100 mg/mL streptomycin. The cells were grown at 37°*C* in 5% CO_2_ incubator. pC-Nluc EBOV VP40 and pC-FLAG-EBOV VP40 have been previously described (Urata et al., 2022).

**Cell toxicity.** The viability of 293T cells after treatment with the compounds was assessed using a CellTiter-Glo Luminescent Cell Viability Assay (G7572, Promega, Madison, WI, USA), which determines the number of viable cells in a culture based on ATP levels. 293T cells (3 × 10^4^ cells/well) were seeded in 96-well plates (167008, Thermo Fisher Scientific, Waltham, MA, USA) after treatment with poly-L-lysine (P4832, Sigma, Burlington, MA, USA) to form a monolayer. One day post-seeding, the cells were treated with compounds (100 μM) or Dimethyl sulfoxide (DMSO, 0.1%) as a control. At 24 h post-treatment, the culture supernatant was removed, and CellTiter-Glo Reagent was added. The assay was performed according to the manufacturer’s recommendations using a SpectraMAX iD5 microplate reader (Molecular Devices, San Jose, CA, USA). The viability of DMSO-treated control cells is set to 1.0, and the relative cell viabilities are shown. Experiments were performed with four independent trials.

***In vitro* screening using Nluc-EBOV VP40.** Screening was performed as described previously (Urata et al., 2022). Briefly, the 293T cells (3 × 10^4^) were seeded in a 96-well plate (167008, Thermo Fisher Scientific), which was coated with poly-L-lysine, and incubated overnight at 37°*C* in 5% CO_2_. The next day, 293T cells were transfected with 0.1 μg of VP40 plasmid using TransIT-LT1 (3 µL TransIT-LT1/µg DNA) (V2305-T, Takara, Shiga, Japan). Culture media were replaced with fresh medium containing compounds (NUEbo) after 5–6 h of transfection and incubated. The following day, the supernatants were separated from the cells, and both were lysed using the Nano-Glo Assay Reagent (Nano-Glo Luciferase assay system, Promega, N1120). After incubation for 10–30 min at room temperature, each sample was transferred to a white 96-well plate (475523, Thermo Fisher Scientific) and luminescence was measured using a SpectraMAX iD5 microplate reader. Experiments were performed with four independent trials.

**50% inhibitory concentration (IC_50_), 50% cytotoxic concentration (CC_50_), and selectivity index (SI) of the selected compounds.** IC_50_ and CC_50_ were determined using the same procedure as described above (Cell toxicity and *In vitro* screening using Nluc-EBOV VP40) using 0 (DMSO), 12.5, 25, 50, 100, and 200 μM of the NUEbo 18, 30, 38, 40, 45, and NUSU#1. SI was also calculated (CC_50_/IC_50_). Experiments were performed with four independent trials.

**VLP assay.** The 293T cells (1.5 × 10^5^) were transfected with 0.5 µg of EBOV VP40 expression plasmid using TransIT-LT1 (2 µL TransIT-LT1/µg DNA). At 6 h post transfection (p.t.), each compound was added to the medium at a final concentration of 100 μM. At 24 h p.t., the VLP-containing culture supernatants and cells were collected. After removing the cell debris using centrifugation (13,000 × *g*; 10 min), VLPs were collected using ultracentrifugation (345,000 × *g*; 30 min at 4*°*C) through a 20% sucrose cushion. Cells and VLP were resuspended in lysis buffer (1% NP-40, 50 mM Tris-HCl [pH 8.0], 62.5 mM EDTA, and 0.4% sodium deoxycholate) and phosphate-buffered saline (PBS) (−), respectively. Resuspended samples were then mixed with SDS sample buffer, treated at 95°C for 5 min, and used for sodium dodecyl sulfate-polyacrylamide gel electrophoresis (SDS-PAGE) and western blotting (WB). Experiments were performed with three independent trials.

**WB.** Cell lysates or VLP samples were resolved using SDS-PAGE gel for VP40 and Actin, followed by WB using an anti-FLAG monoclonal antibody (F1804, Sigma-Aldrich) and anti-actin mouse monoclonal antibody (AC-15, Sigma-Aldrich), respectively. A horseradish peroxidase-conjugated anti-mouse IgG antibody was used as a secondary antibody (A-2304, Sigma-Aldrich). ECL Prime (GE Healthcare, Chicago, IL, USA) and iBright 1500 (Invitrogen) were used to detect the proteins. Band intensities were quantified using ImageJ software.

**Statistical analysis.** GraphPad Prism 5 (GraphPad Software, Inc., San Diego, CA, USA) was used for all statistical analyses. Quantitative data were presented as the mean ± standard deviation (SD) from at least three independent experiments. For all calculations, *p* < 0.05, *p* < 0.01, and *p* < 0.001 are considered significant and are represented by asterisks (*, **, and ***, respectively). Group comparisons are performed using one-way analysis of variance (ANOVA), followed by Dunnett’s multiple comparison test. Welch’s t-test was used to compare two groups.

## 3 Results

**Fig. 1:**
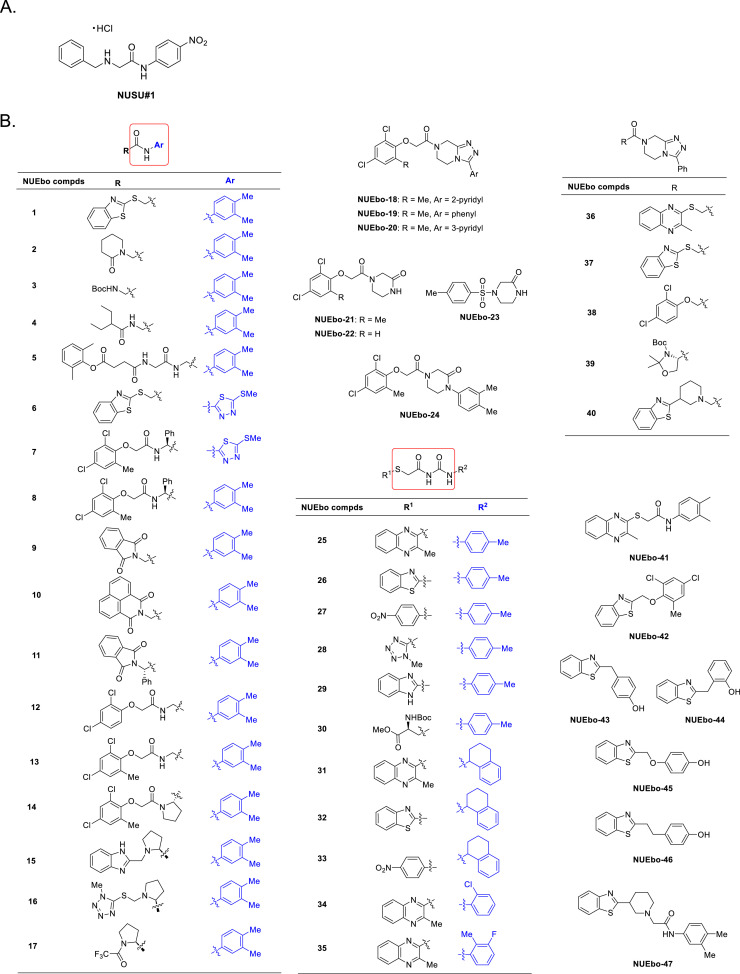
A. Chemical structure of NUSU#1. B. List of chemical structures that were initially planned to be synthesized.

**Fig. 2:**
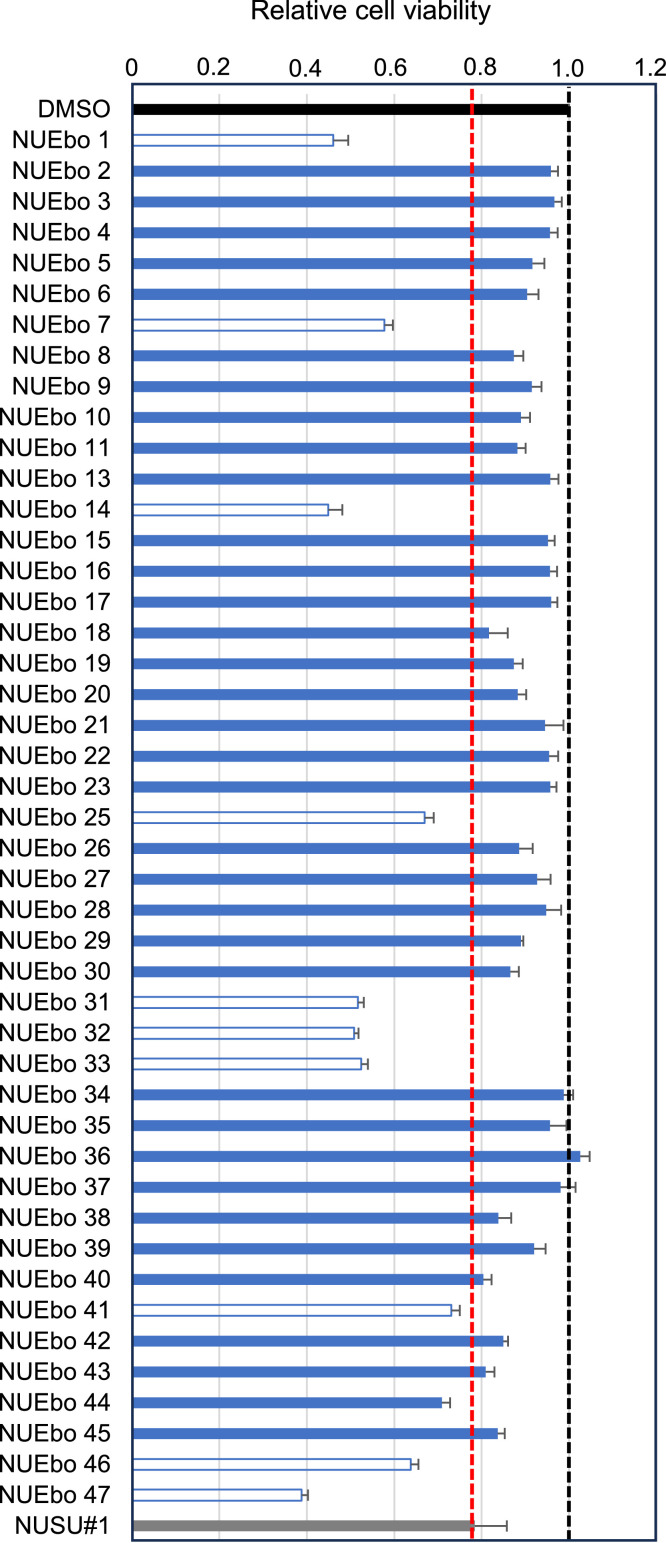
Cell viabilities of NUEbo compounds on 293T cells. 293T cells were seeded in a 96-well plate and treated with each compound (100 μM). Cell viability was measured as described in the materials and methods. Cell viability of DMSO is set as 1.0 (black bar), and that of NUSU#1 is indicated in the gray bar. The blue bars are the samples that exhibited higher cell viability than the NUSU#1. The unfilled bars with the blue line are the samples that exhibited lower cell viability than the NUSU#1 (red dashed line). The average and SD from four independent experiments are shown. (For interpretation of the references to color in this figure legend, the reader is referred to the web version of this article.).

**Ten out of forty-five newly synthesized compounds were excluded due to the higher cell toxicity than NUSU#1.** This study aimed to identify compounds with lower cytotoxicity and higher antiviral activity than those of NUSU#1. Forty-seven chemical compounds were planned to be synthesized from NUSU#1 (Fig. 1A), which we identified as possessing anti-filovirus VP40-mediated VLP production. NUEbo 12 and NUEbo 24 could not be synthesized. Therefore, the viability of forty-five compounds (Fig. 1B), together with DMSO and NUSU#1 as controls, was examined (Fig. 2). 293T cells were mixed with 100 μM compounds (DMSO 0.1%), and the cell viability was examined after 24 h incubation. The cell viability of DMSO is set as 1.0 (black bar), and the relative cell viabilities are shown. Relative cell viability after NUSU#1 treatment is shown as a gray bar. Ten (NUEbo 1, 7, 14, 25, 31, 32, 33, 41, 46, and 47) of the forty-five chemical compounds showed lower cell viability than NUSU#1; therefore, these compounds were excluded from subsequent experiments (Fig. 2).

**Fig. 3:**
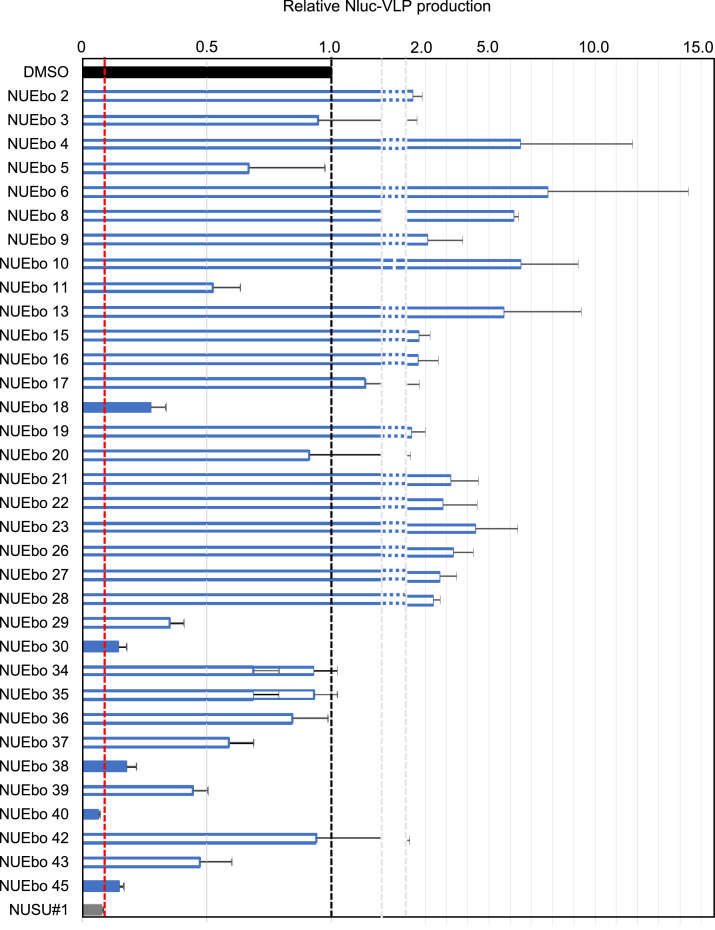
Screening of the selected NUEbo compounds on the EBOV VP40-mediated VLP production using Nluc-EBOV VP40 system. The 293T cells seeded in a 96-well plate were transfected with Nluc-EBOV VP40 expression plasmid (pC-Nluc-EBOV VP40). Culture media was replaced with fresh media containing DMSO, NUEbo, or NUSU#1 (100 μM, 0.1%). The expression of Nluc in both the culture media and the cells was measured. The relative VLP production calculated by the VLP/Cell (Nluc) is shown. VLP/Cell treated with DMSO is set as 1.0 (black bar), and that of NUSU#1 is indicated in the gray bar (Red dashed line). The blue bars are the samples that exhibited less than a 30% reduction of the VLP production compared to that of DMSO treatment. The unfilled bars with a blue line are the samples that are not further analyzed in this study due to the less VLP production inhibitory effect. The average and the SD from four independent experiments are shown. (For interpretation of the references to color in this figure legend, the reader is referred to the web version of this article.).

**Five compounds were selected as hit compounds from the screening.** Next, to evaluate the inhibitory effect of selected chemical compounds from the previous experiment (Fig. 2), a screening system that we developed and reported previously using NanoLuciferase (Nluc)-tagged EBOV VP40 was used (Urata et al., 2022). 293T cells were transfected with Nluc-EBOV VP40 expression plasmid and replaced with the fresh culture media containing each compound (100 μM in DMSO 0.1%). Luminescence from the supernatant and cells was measured and used to calculate the relative VLP production (VLP/cell) compared to the DMSO control treatment. The relative VLP production from the NUSU#1 treated samples is 0.08. In this study, compounds with a relative VLP production of 0.3 or less were selected as hit compounds. The relative Nluc-VLP production of NUEbo 18, 30, 38, 40, and 45 is 0.27, 0.14, 0.17, 0.06, and 0.14, respectively; therefore, they were selected as hit compounds (Fig. 3).

NUEbo 2, 4, 6, 8, 9, 10, 13, 15, 16, 19, 21-28 showed increased VLP production compared to the DMSO treatment, and this was due to the low Nluc expression in the cells. To determine the 50% inhibitory concentration (IC_50_) and 50% cytotoxic concentration (CC_50_), the experiments with the same procedure using multiple compound concentrations (0, 12.5, 25, 50, 100, and 200 μM) were conducted. The IC_50_ of NUEbo 18, 30, 38, 40, 45, and NUSU#1 was 176.6, 29.7, 58.6, 32.6, 73.6, and 39.2 μM, respectively. The CC_50_ of all compound tested was higher than 200 μM. Accordingly, the SI of NUEbo 18, 30, 38, 40, 45, and NUSU#1 is > 1.13, > 6.73, > 3.41, > 6.13, > 2.71, and > 5.10, respectively.

**Fig. 4:**
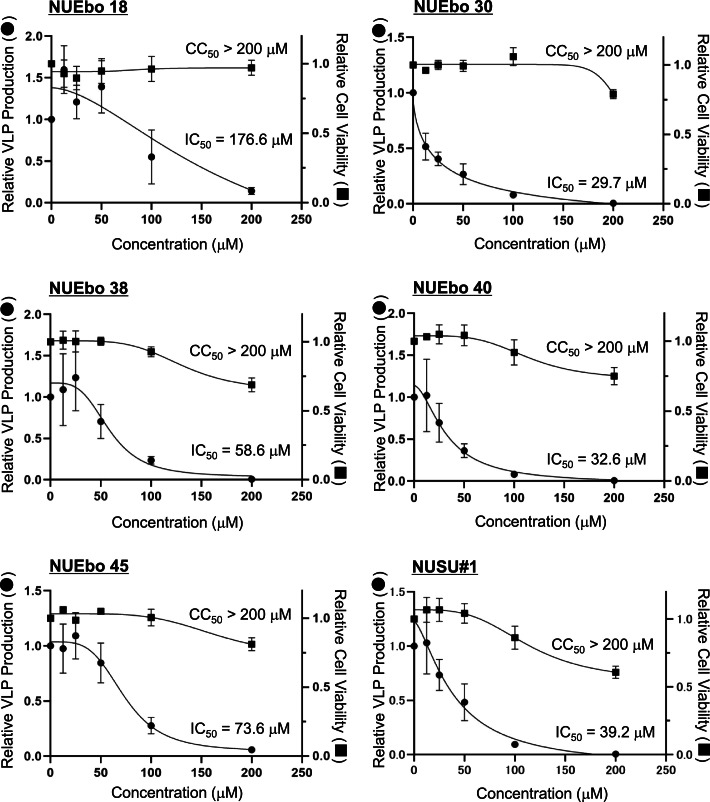
Dose-dependent analysis of the selected compounds on the inhibitory and cytotoxic effects. The 293T cells seeded in a 96-well plate were transfected with Nluc-EBOV VP40 expression plasmid (pC-Nluc-EBOV VP40). Culture media was replaced with fresh media (NUEbo or NUSU#1, 0-200 μM, 0.1%). The expression of Nluc in both the culture media and the cells was measured. The relative VLP production calculated by the VLP/Cell (Nluc) is shown. VLP/Cell treated with DMSO is set as 1.0. The average and the SD from four independent experiments are shown.

**Fig. 5:**
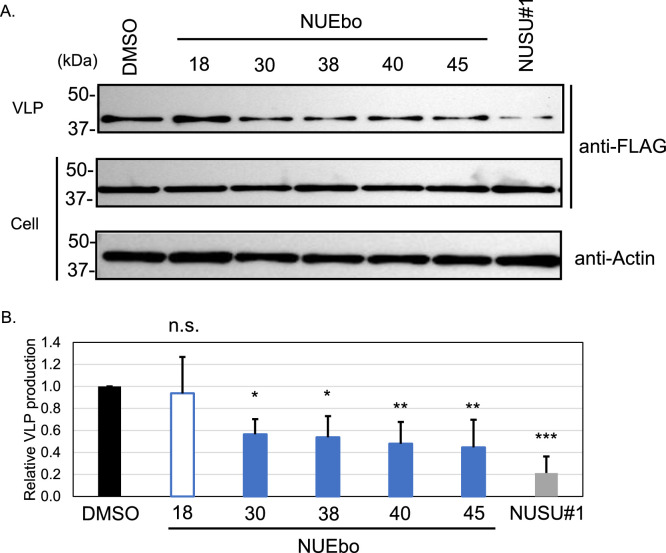
Effect of the selected NUEbo compounds on the VLP production. (A) The VLP assay, was performed against the selected NUEbo compounds. 293T cells were transfected with the FLAG-EBOV VP40 expression plasmid (pC-FLAG-EBOV VP40). At 6 h p.t., DMSO, NUEbo compounds, or NUSU#1 was applied to the media (final 0.1%, 100 μM). At 24 h p.t., the culture media were collected, and ultracentrifugation was performed to concentrate the VLPs. The samples for SDS-PAGE were prepared from the VLP and cell lysate. EBOV VP40 was detected in the VLPs and cell lysate using an anti-FLAG antibody. Actin was also detected in the cell lysate. (B) Quantification and calculation of the relative VLP production measured by the VLP assay in (A). The average and SD from more than three independent experiments are shown. The relative VLP production from the DMSO treatment is set as 1.0 (black bar). The relative VLP production from the NUSU#1 treatment is shown in the gray bar. The samples which showed the reduction of the VLP production with and without statistical significance are shown in filled blue bar and blue lined bar, respectively (*p < 0.05, **p< 0.01, ***p < 0.001). (For interpretation of the references to color in this figure legend, the reader is referred to the web version of this article.).

**VLP assay to validate the effect of the selected compounds.** To evaluate the effects of the selected compounds (Fig. 3, Fig. 4) on VLP production, VLP assay was performed (Urata et al., 2007; Urata et al., 2022; Urata and Yasuda, 2010; Yasuda et al., 2003). 293T cells were transfected with FLAG-tagged EBOV VP40, and each compound was added to the medium. At 24 h p.t., culture supernatants and cell lysates were collected. The culture supernatant was used for ultracentrifugation to pellet down the VLPs. Both VLP and cell samples were subjected to SDS-PAGE, followed by WB to detect EBOV VP40 (Fig. 5A). Relative VLP production was calculated based on the band intensities of VLP and cells (VLP/cell) (Fig. 5B). The relative VLP production of NUEbo 18, 30, 38, 40, and 45 compared to that of DMSO is 0.72, 0.46, 0.38, 0.47, and 0.34, respectively. The reduction in VLP production by NUEbo 30, 38, 40, and 45 compared to that by DMSO is statistically significant, but the reduction efficiency does not reach that of NUSU#1 (0.23).

## 4 Discussion

This study aimed to identify novel chemical compounds derived from NUSU#1 that target VLP production (Urata et al., 2022). Notably, NUSU#1 inhibited not only EBOV but also BDBV, SUDV, TAFV, RESTV, and MARV VP40-mediated VLP production. To identify novel chemical compounds with low cytotoxicity and high activity, we prepared a class of amides, carbamoyl acetamides, and other derivatives based on NUSU#1. Using the cell viability assay, our previously developed screening assay (Urata et al., 2022), and the VLP assay, four novel compounds (NUEbo 30, 38, 40, and 45) that inhibited EBOV VP40-mediated VLP production were identified. The inhibitory effects from the screening assay and the VLP assay do not completely correspond, and this could be explained by the different procedure to quantify the VLP production. The purpose of the screening is to select the hit candidates quickly; therefore, there is no purification step. In contrast, there is a purification step of the VLP using the ultracentrifuge with 20% sucrose in the VLP assay. Although none of these compounds exhibited a stronger inhibitory effect than the original compound, NUSU#1, at the same concentration (100 μM), they showed less cell toxicity, suggesting their potential as a resource for further chemical synthesis to identify better chemical compounds. In the screening assay, some compounds (NUEbo 2, 4, 6-10, 13-16, 21-28) exhibited a significant increase of the Nluc-VLP production (Fig. 3), and this was due to the significant reduction in the Nluc expression in the cell, but not due to the cell toxicity (Fig. 2). Further experiment would be required to understand this reason. Consistent to the results in Fig. 2, NUEbo 30, 38, 40, and 45 did not exhibit significant cell toxicities based on the expressions of Actin and EBOV VP40 in the cells (Fig. 5A). There are no clear common structural features among these four hits that might correspond to the inhibitory effect. Although no direct evidence exists, NUSU#1 was identified through an *in silico* screening targeting the interface of the EBOV VP40 dimer. Further experiments would be required to identify the precise targets of these selected compounds.

Among the compounds synthesized in this study, NUEbo 25 was almost equivalent to compound 5 reported in a previous study (Han et al., 2014). Compound 5 inhibits EBOV VP40, MARV VP40, and Lassa virus Z-mediated VLP production (Han et al., 2014). The only difference between NUEbo 25 and the phenylcarbamoyl acetamide derivative, compound 5, was the substitution of a methyl group on the phenyl ring. In our study, NUEbo 25 was excluded from the first step because of its higher cell toxicity than that of NUSU#1. Based on this result, it may be worth examining compounds at a lower concentration than that tested in this study (100 μM). In addition, NUEbo 34 and 35 are also composed of an arylcarbamoyl acetamide scaffold, with the difference being the presence of chloride (NUEbo 34) and methyl and fluorine (NUEbo 35) on the phenyl ring. None of them showed a significant inhibitory effect on EBOV VP40-mediated VLP production.

Filovirus VP40 is a multitasking viral protein that is regulated by forming different conformations by interacting with not only the PM (Bennett et al., 2020), but also a variety of host and viral factors, and some of their conformations have been reported (Landeras-Bueno et al., 2021; Wan et al., 2020; Watanabe et al., 2024). A recent study also described the differences in the structures of EBOV and SUDV VP40 (Clifton et al., 2015; Werner et al., 2023), especially at the C-terminus, further confirming the importance of understanding the common and different features among virus species and identifying novel chemical compounds that could broadly target human pathogenic filoviruses or develop virus-specific therapeutics. In addition, combinations targeting both particle production and other viral replication steps in the cell could exhibit additive or synergetic effects that might be worth examining in future studies. Refering the selected compounds identified in this study and the screening system we developed and used here or recently reported HiBiT (33 bp, 11 aa)-based system (Furuyama et al., 2025) would facilitate to identify novel compounds targeting filovirus VP40-mediated VLP production.

## 5 Conclusion

Through the screening assay we developed previously using forty-five novel synthesized chemical compounds derived from the NUSU#1, the novel chemical compound that showed significant reduction of the EBOV VP40-mediated VLP production, followed by the VLP assay, four compounds were identified to exhibit the reduction of the EBOV VP40-mediated VLP production with the less cell toxicity compared to the NUSU#1.

## Funding information

This work was supported by the Japan Agency for Medical Research and Development (AMED) (JP24fm0208101, JP25fm0208101, and 24jk0210001h0001), the research grant of Astellas Foundation for Research on Metabolic Disorders, Kanae Foundation for the Promotion of Medical Sciences to SU.

## CRediT authorship contribution statement

**Tomoko Tsuruta:** Writing – review & editing, Investigation. **Satoshi Mizuta:** Writing – review & editing, Visualization, Methodology, Investigation, Formal analysis. **Ayako Izumisawa:** Investigation. **Jiro Yasuda:** Resources, Funding acquisition. **Shuzo Urata:** Writing – review & editing, Writing – original draft, Visualization, Validation, Supervision, Resources, Methodology, Investigation, Funding acquisition, Formal analysis, Data curation, Conceptualization.

## Declaration of competing interest

The authors declare that they have no competing financial interests or personal relationships that may have influenced the work reported in this study.

## Data Availability

Data will be made available on request.

## Appendix Supplementary materials

Image, application 1
