# Supplementary material for: Identification of novel small chemical compounds that inhibit Ebola virus VP40-mediated virus-like particle production
Source: Virus Res. 2026 Jan 3;364:199684. doi: 10.1016/j.virusres.2026.199684 (PMC12818169; doi:10.1016/j.virusres.2026.199684)
Supplement: Supplementary file 1 [file mmc1.docx]

**Supplemental Information**

1. **General Information**

All nuclear magnetic resonance (NMR) spectra were recorded on a Varian 500PS spectrometer or JEOL JMN-ECZ400R. ^1^H, ^13^C and ^19^F NMR spectra were reported as chemical shifts (δ) in parts per million (ppm) relative to the solvent peak using tetramethylsilane (^1^H and ^13^C) as an internal standard. Chemical shifts (δ) are quoted in parts per million (ppm), and coupling constants (*J*) are measured in hertz (Hz). The following abbreviations were used to describe multiplicities: s = singlet, d = doublet, t = triplet, q = quartet, quint. = quintet, br = broad, m = multiplet. The NMR spectra were processed using ACD/SpecManager. High-resolution mass spectra (HRMS, *m*/*z*) were obtained on a JEOL JMS-700N for fast atom bombardment (FAB) using m-nitrobenzyl alcohol as a matrix. All the reactions were performed in an apparatus with magnetic stirring under an inert atmosphere. Flash column chromatography was performed using Fuji Silysia Chemical Ltd. Silica Gel C60 (50–200 μm) using an eluent system, as described in the experimental section, i.e., Experimental Procedures. Thin*-*layer chromatography was performed using TLC Silica Gel 60 F254 aluminum sheets (Merck) and Silica Gel F254 glass plates (Merck).

**2. Experimental procedures and characterization data**

**Synthesis of 2-(2,4-dichloro-6-methylphenoxy)-1-(3-(pyridin-2-yl)-5,6-dihydro-[1,2,4]triazolo**

**[4,3-a] pyrazin-7(8H)-yl) ethan-1-one (NUEbo 18)**

A 20 mL round-bottom flask quipped with a magnetic stirring bar was charged with 3-(pyridin-2-yl)-5,6,7,8-tetrahydro [1,2,4] triazolo[4,3-a] pyrazine (40 mg, 0.2 mmol), 2-(2,4-dichloro-6-methylphenoxy) acetic acid (47 mg, 0.2 mmol), 1-hydroxybenzotriazole (2.7 mg, 0.02 mmol), 1-(3-Dimethylaminopropyl)-3-ethylcarbodiimide Hydrochloride (EDCI) (38 mg, 0.2 mmol), diisopropylethylamine (71 μL, 0.4 mmol) and DMF (1.5 mL). The resulting mixture was stirred overnight. Deionized H_2_O was added to the reaction mixture and was extracted with EtOAc. The organic layers were washed with brine, dried over MgSO_4,_ and evaporated in vacuo. The residue was purified by column chromatography, eluting with EtOAc−MeOH (10:1). This afforded the title compound as a white solid (39 g, 47 % yield).

**^1^H NMR** (500 MHz, CDCl_3_): δ 8.67−8.58 (m, 1H), 8.37−8.29 (m, 1H), 7.84 (dt, *J* = 1.5, 7.9 Hz, 1H), 7.39−7.31 (m, 1H), 7.29−7.22 (m, 1H), 7.11 (d, *J* = 8.8 Hz, 1H), 5.22 (s, 1H), 5.13 (s, 1H), 4.83−4.65 (m, 4H), 4.27−4.19 (m, 1H), 4.17−4.10 (m, 1H), 2.31 (s, 3H); **^13^C{^1^H} NMR** (125 MHz, CDCl_3_): δ 166.6, 166.3, 151.5−151.2 (m), 149.0−148.5 (m), 147.6, 147.5, 137.1, 134.6, 134.4, 130.3, 130.0, 129.8, 128.1, 127.8, 127.7, 124.1, 123.0, 72.2, 71.4, 46.3, 45.3, 42.8, 50.4, 39.4, 39.4, 29.7, 16.5; HRMS (FAB) *m/z* Calcd for C_19_H_18_Cl_2_N_5_O_2_ [M+H]^+^ 418.0838 found 418.0838.

**Synthesis of methyl N-(tert-butoxycarbonyl)-S-(2-oxo-2-(3-(p-tolyl) ureido) ethyl)-L-cysteinate (NUEbo 30)**

To a solution of methyl (tert-butoxycarbonyl)-L-cysteinate (59 mg, 0.25 mmol) in EtOH (1.2 mL) was added 2-chloro-N-(p-tolylcarbamoyl) acetamide (50 mg, 0.25 mmol), and AcONa (32 mg, 0.38 mmol). The mixture was stirred at 80 ℃ overnight. After the reaction, the reaction mixture was cooled to room temperature. The precipitate was filtered off, washed with EtOH. The filtrate was dried under reduced pressure and afforded the title compound as a white solid (20 mg, 19 % yield) without further purification.

**^1^H NMR** (500 MHz, CDCl_3_): δ 10.3 (br.s, NH), 9.39 (br.s, NH), 7.39 (d, *J* = 8.6 Hz, 2H), 7.14 (d, *J* = 8.1 Hz, 2H), 5.45 (d, *J* = 7.6 Hz, 1H), 4.59 (br.s, NH), 3.80 (s, 3H), 3.40 (d, *J* = 2.0 Hz, 2H), 3.14 (dd, *J* = 4.7, 13.9 Hz, 1H), 3.02 (dd, *J* = 5.6, 13.9 Hz, 1H), 2.33 (s, 3H), 1.46 (s, 9H); **^13^C{^1^H} NMR** (125 MHz, CDCl_3_): δ 171.1, 1701, 55.2, 150.7, 134.2, 129.5, 120.4, 80.6, 53.1, 552.9, 36.7, 35.3, 28.3, 20.8; HRMS (FAB) *m/z* Calcd for C_19_H_28_N_3_O_6_S [M+H]^+^ 426.1699 found 426.1699.

**Synthesis of N-((3-fluoro-2-methylphenyl) carbamoyl)-2-((3-methylquinoxalin-2-yl) thio) acetamide (NUEbo-35)**

To a solution of 3-methylquinoxaline-2-thiol (44 mg, 0.25 mmol) in EtOH (1.2 mL) was added 2-chloro-N-(p-tolylcarbamoyl) acetamide (50 mg, 0.25 mmol), and AcONa (32 mg, 0.38 mmol). The mixture was stirred at 80 °C overnight. After the reaction, the reaction mixture was cooled to room temperature. The precipitate was filtered off, washed with EtOH. The filtrate was dried under reduced pressure and afforded the title compound as a white solid (55 mg, 57% yield) without further purification.

**^1^H NMR** (400 MHz, CDCl_3_): δ 10.3 (br.s, NH), 10.2 (br.s, NH), 8.06 (d, *J* = 8.1 Hz, 1H), 8.02 (d, *J* = 8.1 Hz, 1H), 7.77 (d, *J* = 8.3 Hz, 1H), 7.76−7.69 (m, 2H), 7.12 (q, *J* = 7.9 Hz, 1H), 6.83 (t, *J* = 8.8 Hz, 1H), 4.07 (s, 2H), 2.75 (s, 3H), 2.23 (s, 3H); **^13^C{^1^H} NMR** (125 MHz, DMSO-d_6_): δ 171.6, 160.1 (d, *J* = 238.7 Hz), 154.6, 151.6, 150.9, 140.4, 139.1, 137.7 (d, *J* = 6.6 Hz), 129.9, 128.9, 128.4, 127.3 (d, *J* = 9.5 Hz), 127.0, 117.3, 115.3 (d, *J* = 19.0 Hz), 110.8 (d, *J* = 21.8 Hz), 34.5, 21.8, 9.05 (d, *J* = 5.7 Hz); **^19^F NMR** (376 MHz, CDCl_3_): δ −115.6 (m, 1H); HRMS (FAB) *m/z* Calcd for C_19_H_18_FN_4_O_2_S [M+H]^+^ 385.1134 found 385.1134.

**2-(2,4-dichlorophenoxy)-1-(3-phenyl-5,6-dihydro-[1,2,4] triazolo[4,3-a]pyrazin-7(8H)-yl)ethan-1-one (NUEbo 38)**

A 20 mL round-bottom flask quipped with a magnetic stirring bar was charged with 3-phenyl-5,6,7,8-tetrahydro [1,2,4] triazolo[4,3-a] pyrazine (88 mg, 0.4 mmol), 2-(2,4-dichlorophenoxy) acetic acid (80 mg, 0.4 mmol), 1-hydroxybenzotriazole (5.4 mg, 0.04 eq), 1-(3-Dimethylaminopropyl)-3-ethylcarbodiimide Hydrochloride (EDCI) (77 mg, 0.4 mmol), diisopropylethylamine (143 μL, 0.8 mmol) and DMF (3.0 mL). The resulting mixture was stirred overnight. Deionized H_2_O was added to the reaction mixture and was extracted with EtOAc. The organic layers were washed with brine, dried over MgSO_4,_ and evaporated in vacuo. The residue was purified by column chromatography, eluting with EtOAc−MeOH (10:1). This afforded the title compound as a white solid (84 g, 52 % yield).

**^1^H NMR** (500 MHz, DMSO-d_6_): δ 7.78−7.69 (m, 1H), 7.56 (d, *J* = 6.4 Hz, 3H), 7.32 (d, *J* = 8.8 Hz, 1H), 7.14 (t, *J* = 8.8 Hz, 1H), 5.17 (s, 2H), 4.98 (s, 1H), 4.87 (s, 1H), 4.32−4.23 (m, 1H), 4.18−4.08 (m, 1H), 3.94−3.85 (m, 2H); **^13^C{^1^H} NMR** (125 MHz, DMSO-d_6_): δ 166.3, 166.2, 152.7−152.5 (m), 148.3, 148.2, 130.0, 129.3, 120.1, 128.0, 127.9, 126.9, 126.8, 124.8, 124.8, 122.8, 115.5, 66.5, 66.4, 43.8, 43.4, 41.2−41.1 (m), 38.6; HRMS (FAB) *m/z* Calcd for C_19_H_17_Cl_2_N_4_O_2_ [M+H]^+^ 403.0729 found 403.0729.

**2-(3-(benzo[d]thiazol-2-yl) piperidin-1-yl)-1-(3-phenyl-5,6-dihydro [1,2,4] triazolo[4,3-a]pyrazin-7(8H)-yl)ethan-1-one (NUEbo 40)**

To a solution of 2-chloro-1-(3-phenyl-5,6-dihydro [1,2,4] triazolo[4,3-a] pyrazin-7(8H)-yl) ethan-1-one (65 mg, 0.3 mmol) in DMF (2.5 mL) was added 2-(piperidin-3-yl) benzo[d]thiazole (83 mg, 0.3 mmol), and K_2_CO_3_ (83 mg, 0.6 mmol). The mixture was stirred at 80 ℃ overnight. The reaction mixture was cooled to room temperature and diluted with deionized H_2_O. The resulting solution was extracted with EtOAc, washed with brine, dried over MgSO_4,_ and evaporated in vacuo. The residue was purified by column chromatography, eluting with EtOAc−MeOH (4:1). This procedure afforded the title compound as a white solid (71 mg, 52 % yield).

**^1^H NMR** (500 MHz, CDCl_3_): δ 7.95−7.90 (m, 1H), 7.81−7.78 (m, 1H), 7.66−7.64 (m, 1H), 7.58−7.54 (m, 1H), 7.49−7.46 (m, 3H), 7.44−7.38 (m, 1H), 7.30 (t, *J* = 7.3 Hz, 1H), 5.29−4.98 (m, 2H), 4.13−3.81 (m, 4H), 3.52−3.33 (m, 3H), 3.24−3.08 (m, 1H), 2.97−2.58 (m, 2H), 2.52−2.33 (m, 1H), 2.22−2.10 (m, 1H), 1.96−1.67 (m, 3H); **^13^C{^1^H} NMR** (125 MHz, CDCl_3_): δ 173.2, 169.0, 153.4, 153.1, 152.7, 148.4, 148.2, 134.4, 134.4, 130.2, 130.0, 129.0, 128.0, 127.9, 126.2 (m), 126.1, 125.9, 124.9, 124.7, 122.5, 122.4, 121.6, 121.5. 62.1, 61.8, 53.7, 53.3, 44.3, 43.6, 43.2, 42.7, 41.2, 40.1, 39.2, 20.2, 29.6, 24.3−24.1 (m), HRMS (FAB) *m/z* Calcd for C_25_H_27_N_6_OS [M+H]^+^ 459.1967 found 459.1966.

1. **NMR data**

Figure S1. ^1^HNMR of **NUEbo 18** (500 MHz, CDCl_3_)

Figure S2. ^13^CNMR of **NUEbo 18** (125 MHz, CDCl_3_)

Figure S3. ^1^HNMR of **NUEbo 30** (500 MHz, CDCl_3_)

Figure S4. ^13^CNMR of **NUEbo 30** (125 MHz, CDCl_3_)

Figure S5. ^1^HNMR of **NUEbo 35** (400 MHz, CDCl_3_)

Figure S6. ^13^CNMR of **NUEbo 35** (125 MHz, DMSO-d_6_)

Figure S7. ^19^FNMR of **NUEbo 35** (376 MHz, CDCl_3_)

Figure S8. ^1^HNMR of **NUEbo 38** (500 MHz, DMSO-d_6_)

Figure S9. ^13^CNMR of **NUEbo 38** (125 MHz, DMSO-d_6_)

Figure S10. ^1^HNMR of **NUEbo 40** (500MHz, CDCl_3_)

Figure S11. ^13^CNMR of **NUEbo 40** (125MHz, CDCl_3_)
